# Physiological responses to short‐term high‐altitude acclimatization: Insights from predictive modeling approaches

**DOI:** 10.14814/phy2.70711

**Published:** 2026-01-05

**Authors:** Valeria Páez, Sofia Lozano, Danixza Calfil, David Cristóbal Andrade, Maria Rodriguez‐Fernandez

**Affiliations:** ^1^ Institute for Biological and Medical Engineering Pontificia Universidad Católica de Chile Santiago Chile; ^2^ Exercise Applied Physiology Laboratory, Biomedical Department, Faculty of Health Sciences Universidad de Antofagasta Antofagasta Chile

**Keywords:** baroreflex, chemoreflex, exercise, high altitude, machine learning, performance

## Abstract

High‐altitude (HA) exposure induces cardiovascular, respiratory, and metabolic adjustments that often impair exercise performance. These physiological responses depend on hypoxic severity, exposure duration, and individual susceptibility. Although full acclimatization generally requires about 7 days, early adaptations can emerge within the first 72 h. This study aimed to characterize these early responses and to evaluate the potential of mathematical modeling to predict HA‐related exercise performance decline. Nine healthy volunteers (age: 24.4 ± 3.3; weight: 63.7 ± 11.8; height: 169.4 ± 8.4; female: 44%) completed maximal cardiopulmonary exercise tests under three conditions: at sea level (SL), and at 3015 m after 12 h (HA12h) and 60 h (HA60h) of exposure. Although 60 h at HA was insufficient for full acclimatization, significant differences were observed between HA12h and HA60h, indicating partial physiological adaptation. Maximal power output declined at both HA time points. Notably, HA‐induced performance deterioration was accurately predicted (*R* ^2^ = 0.81) using SL‐derived parameters, particularly maximal oxygen pulse (*VO*_2_/*HR*_max_) and the ventilatory equivalent for carbon dioxide (*VE*/*VCO*_2_). These findings provide novel insights into early physiological responses to HA and support the development of individualized, model‐based tools to anticipate performance loss and optimize training and acclimatization strategies.

## 1 INTRODUCTION

High‐altitude (HA) exposure induces several short‐ to long‐term physiological adaptations, including cardiovascular, respiratory, metabolic, autonomic, hematological, and endocrine changes (Alvarez‐Araos et al., 2024; Richalet et al., 2024; Tymko et al., 2023). These responses are triggered by the progressive decline in barometric pressure and in the partial pressure of oxygen in the inhaled air (*PiO*_2_) with increasing altitude. This reduction in *PiO*_2_ leads to lower arterial oxygen saturation (*S*p*O*_2_) and consequently diminished oxygen availability at the tissue level (Richalet et al., 2024).

The altitude level and duration of hypoxic exposure play a crucial role in determining individual physiological responses to HA (Tymko et al., 2023). While proper acclimatization to hypoxia is generally achieved after 7–10 days (Mallet et al., 2023; Richalet et al., 2024), some authors have reported earlier acclimatization occurring in 5 days (Purkayastha et al., 1995) or even during brief exposures of ≤3 days (Muza et al., 2010; Sharma et al., 2023). However, short‐term acclimatization mechanisms remain poorly understood (Alvarez‐Araos et al., 2024), particularly regarding the integration or differentiation of cardiovascular, autonomic, and metabolic response during the initial hours or days of HA exposure (Steinback et al., 2009).

A prominent effect of HA is the decline in exercise performance, a critical issue in the context of sports science, tourism, and high‐altitude occupations such as mining. Traditionally, this performance loss has been quantified using the maximal oxygen uptake (*VO*_2max_) (Alvarez‐Araos et al., 2024; Townsend et al., 2017; Wehrlin & Hallén, 2006). However, recent studies have expanded assessment metrics to include time trials, work capacity, endurance time, exercise economy, maximal power output, and lactate threshold (Chapman et al., 2016; Clark et al., 2007; Páez et al., 2023; Pinilla & Cecilia, 2014; Townsend et al., 2017). These parameters reflect both central and peripheral fatigue from the locomotor system (Amann et al., 2020; Townsend et al., 2017), where muscle afferent feedback influences central motor drive (Alvarez‐Araos et al., 2024; Gandevia, 1998; Townsend et al., 2017). Among these, maximal power output is a robust functional marker of neuromuscular efficiency (Davies & Sandstrom, 1989; Kourou et al., 2015).

The magnitude of exercise performance declines at HA varies widely among individuals, with some experiencing severe deterioration while others only exhibit moderate impairment. Predicting this individual performance deterioration remains a key challenge in the field.

Beyond performance deterioration, reduced oxygen availability at HA also poses serious health risks such as Acute Mountain Sickness (AMS). Classical statistical models have been developed to predict AMS, aiming to prevent life‐threatening events or costly evacuations (Canouï‐Poitrine et al., 2014; Richalet et al., 2012). However, these models are limited in their capacity to predict physical performance at altitudes. Among the limitations of these classical statistical studies are the inability to detect individual susceptibility patterns and the reliance on self‐reported questionnaires (Wang et al., 2024). Since most hypoxia assessments are based on subjective symptoms, a growing push exists to quantify physiological responses (Song et al., 2025).

Recent advances in artificial intelligence (AI), particularly machine learning, have introduced powerful tools for analyzing HA‐related physiological data and predicting HA exercise performance. These models surpass traditional statistical methods in predictive accuracy, generalizability, and the ability to handle large datasets. They can uncover nonlinear relationships and subtle interactions among multiple variables (Chen et al., 2024; Cruz & Wishart, 2007; Kourou et al., 2015; Liu et al., 2022), thereby enabling more accurate and personalized predictions of performance at HA.

In this context, understanding short‐term acclimatization to HA is both scientifically and practically important for reducing the health risks and developing effective acclimatization strategies to optimize physical performance. Therefore, the present study aimed to: (i) investigate the effects of short‐term HA exposure on cardiorespiratory, autonomic, and metabolic responses both at rest and during incremental exercise testing; and (ii) develop preliminary models to predict exercise performance decline at HA based on physiological markers.

## 2 METHODS

### 2.1 Participants

Nine healthy volunteers (5 men, 4 women) were enrolled based on the following inclusion criteria: healthy adults aged 18–50 years, residence below 1000 m, no travel to altitudes above 1500 m in the prior 3 months, no history of metabolic, cardiac, respiratory, or neuromuscular pathologies. Exclusion criteria included hormonal treatment, pregnancy or lactation, severe head or neck trauma, personal or familial history of migraines, and any conditions impairing the execution of the motor task (e.g. inability to cycle). All participants exhibited normal pulmonary function by spirometry (forced expiratory volume in 1 s to forced vital capacity ratio, VEF_1_/FVC >70%), in accordance with a previous study (Bourdillon et al., 2018). Written informed consent was obtained from all participants.

### 2.2 Experimental protocol

**FIGURE 1:**
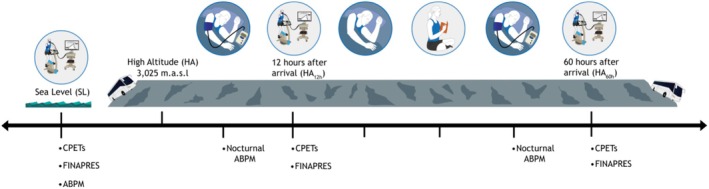
Data Collection Protocol. Participants performed a cardiopulmonary exercise test (CPET) with beat‐to‐beat hemodynamic monitoring and 24‐h Ambulatory Blood Pressure Monitoring (ABPM) under three conditions: Sea level (SL) and after 12 and 60 h of high altitude (HA) exposure.

Data collection was conducted through a field expedition to Valle Nevado, Chile, located at an altitude of 3015 m (Dirección Meteorológica de Chile, 2006). Each participant underwent four measurement sessions: two at sea level (SL), with the first serving as a familiarization session, and two at high altitude following 12 h (HA12h) and 60 h (HA60h) of exposure (Figure 1). All tests at HA were performed in the morning (06:30–12:00). SL tests were conducted at consistent times, 48 h apart. Pre‐test instructions, calibration protocols, and reference values followed established standards (Páez et al., 2023).

### 2.3 Measurements and materials

Participants underwent cardiopulmonary exercise tests (CPET) using a K5 Sports ergospirometer (Cosmed) and a Lode cycle ergometer (Discover Corival CPET). The protocol consisted of four phases: (i) rest (1 min, seated, no pedaling), (ii) warm‐up (1 min at 0 W, 50–55 rpm), (iii) incremental exercise (15 W/min ramp protocol, maintaining 90–95 rpm until volitional fatigue) and (iv) recovery (5 min at 25 W, pedaling at 50–55 rpm). Termination criteria followed standard guidelines (American Thoracic Society & American College of Chest Physicians, 2003). Pre‐test instructions included wearing comfortable clothing and sports shoes, avoiding smoking, alcohol, and vigorous exercise for at least 24 h, and fasting for 2 h.

Environmental conditions were measured using the K5 Sports ergospirometer. At HA12h, the ambient temperature was 21.3°C ± 2.0°C with a relative humidity of 37.0% ± 7.5% and a barometric pressure of 530 ± 0.8 mmHg. At HA60h the temperature was 19.1°C ± 2.4°C with a relative humidity of 33.4% ± 4.8% and a barometric pressure of 530 ± 0.5 mmHg. At SL, the ambient temperature was 24.2°C ± 1.9°C with a relative humidity of 48.3% ± 4.03% and a barometric pressure of 710.4 mmHg.

Baseline measurements included body composition analysis using an InBody 120 analyzer following an 8‐h fast, and pulmonary volumes and capacities, specifically, forced expiratory volume in 1 s (VEF_1_), forced vital capacity (FVC), and VEF_1_ to FVC ratio (VEF_1_/FVC) were assessed using a spirometry pod (PowerLab and Spirometry LabChart Extension).

The respiratory exchange ratio (RER) was assessed through indirect calorimetry, providing insight into the relative contributions of carbohydrates and fats to energy production. Energy expenditure (EE) was calculated according to the Weir Equation (Weir, 1949):

$$\begin{array}{cc} \mathrm{EE}\;\left(\text{kcal}/\mathrm{day}\right)\;=\; & 3.9\times {\mathrm{VO}}_{2}\;\left(L/\min\right)+1.1\\ & \times {\mathrm{VCO}}_{2}\;\left(L/\min\right)\times 1.44 \end{array}$$

Oxygen consumption (*VO*_2_), carbon dioxide production (*VCO*_2_), respiratory exchange ratio (RER), and minute ventilation (VE) were obtained using the OMNIA software's metabolic module. Electrocardiogram (ECG) and continuous blood pressure were monitored using a beat‐to‐beat finger cuff device (Finapres® NOVA). Heart rate (HR) was monitored using a chest strap heart rate monitor (GARMIN, HRM Dual), and *S*p*O*_2_ was measured with a portable finger pulse oximeter (BioRadio™). Blood lactate concentration was measured in duplicate before and after each test using reactive strips with an Accutrend device (Accutrend Plus, Germany).

Hypoxic ventilatory response (HVR) at rest was evaluated using the method described by Richalet et al. (Richalet et al., 2012), following the formula:

$${\mathrm{HVR}}_{r}=\frac{{\mathrm{VE}}_{\text{hypoxia}}-{\mathrm{VE}}_{\text{normoxia}}\;}{∆{\mathrm{Sa}}_{r}\;}/\mathrm{BW}\times 100\;\left(L/\min/\mathrm{kg}\right)$$

where BW is the body weight and $∆{\mathrm{Sa}}_{r}:$

$$∆{\mathrm{Sa}}_{r}={\mathrm{SpO}}_{2\text{normoxia}}-{\mathrm{SpO}}_{2\text{hypoxia}\;\left(\%\right)}$$

To obtain baseline values during the resting protocol, subjects maintained spontaneous breathing for 1 min.

Ambulatory blood pressure monitoring (ABPM) over 24 h was performed on three occasions: SL, HA12h, and HA60h, using the Oscar 2 device from SunTech Medical. The wake–sleep cycle for each subject was determined based on the data from the Fitbit Sense 2 smartwatch. The SL data corresponded to the day prior to the ascent, and the HA data corresponded to each respective day at high altitude.

### 2.4 Statistical analyses

All statistical analyses were conducted in MATLAB (MATLAB R2024a, 2024). Signal preprocessing included phase segmentation by exercise stage (rest, warm‐up, exercise, and recovery) and condition (SL, HA12h, and HA60h). Outliers were removed using a threshold of ±2 standard deviations with a 5‐point moving window or values exceeding twice the mean. For analysis, data were averaged over the final 30 s of each phase. Lactate concentrations were calculated as the average of duplicate measurements taken before and after exercise.

Respiratory exchange ratio (RER) analysis during rest included just 6 subjects, and the hemodynamic variables measured at maximal exercise were excluded due to data noise. HVR was analyzed in 8 subjects; one was excluded due to incomplete *S*p*O*_2_ data at rest. For all participants, data from the second SL test were used, except for one individual from whom *S*p*O*_2_ and CPET data from the first session were analyzed due to missing data from the second session. Data were averaged in one‐min intervals. *S*p*O*_2_ data >100% or <50% were excluded.

For ABPM, outliers were defined as readings by the Suntech software of the OSCAR 2 device as “Air loss” or “Artifact/Irregular signal”, as well as values that deviated by ±20 mmHg from the trend established by three preceding and three following measurements of systolic blood pressure (SBP) or diastolic blood pressure (DBP).

For the calculation of nocturnal dipping, the following formula was applied (Sturgeon et al., 2009; Yang et al., 2015):

$$\mathrm{Dip}\;\mathrm{BP}\;\left(\text{mmHg}\right)=\frac{{\mathrm{MAP}}_{\text{vigil}}-{\mathrm{MAP}}_{\text{sleep}}}{{\mathrm{MAP}}_{\text{vigil}}}\times 100$$

The Wilcoxon signed‐rank test was used to assess whether there were significant changes in the variables between paired conditions (HA and SL). A *p*‐value <0.05 was considered indicative of a statistically significant effect.

### 2.5 Modeling

To predict exercise performance loss at HA, supervised machine learning models were built using MATLAB Regression Learner app (R2024a). The workflow included the following steps:

#### 2.5.1 Data preprocessing

All 40 predictors (Table 1) obtained from measurements at SL—including ergoespirometry (rest: *n* = 10, exercise: *n* = 12), continuous hemodynamics at rest (*n* = 2), ABPM (*n* = 7), and baseline traits (*n* = 9, excluding the categorical variable sex)—were normalized prior to model training.

**TABLE 1:** Summary of sea level (SL) predictors used in the machine learning models. Predictors were grouped into five categories: ergoespirometry during exercise, ergoespirometry at rest, continuous hemodynamics at rest, ambulatory blood pressure monitoring (ABPM), and baseline anthropometric and pulmonary characteristics.

Source	*n*	Variables
Ergoespirometry (exercise)	12	*VE*/*VCO*_2max_, *VO*_2_/*HR*_max_, RF_max_, VE_max_, *VO*_2max_, *VCO*_2max_, RER_max_, HR_max_, METS_max_, *PETCO*_2max_, *PETO*_2MAX_, Time Exercise
Ergoespirometry (rest)	10	HR, METS, *VE*/*VCO*_2_, *VO*_2_, *VCO*_2_, VE, BR, PetCO_2_, PetO2, *VO*_2_/*HR*
Continuous hemodynamics (rest)	2	SBP, DBP
ABPM	7	Blood pressure dip, Mean SBP 24 h, Mean SBP sleep, Mean SBP vigil, Mean DBP 24 h, Mean DBP sleep, Mean DBP vigil
Basal characteristics	9	Age, weight, height, BMI, skeletal muscle mass, body fat mass, FVC, VEF_1_, VEF_1_/FVC

Abbreviations: BMI, body mass index; DBP, diastolic blood pressure; FVC, forced vital capacity; HR, heart rate; METS, metabolic equivalents; *PETCO*_2_, end‐tidal carbon dioxide pressure; *PETO*_2_, end‐tidal oxygen pressure; RER, expiratory exchange ratio; SBP, systolic blood pressure; *VCO*_2_, carbon dioxide production; VE, minute ventilation; *VE*/*VCO*_2_, ventilatory equivalent for carbon dioxide; VEF_1_, forced expiratory volume in 1 s; VEF_1_/FVC, forced expiratory volume in 1 s to forced vital capacity ratio; *VO*_2_, oxygen consumption; *VO*_2_/*HR*_max_, pulse of oxygen.

#### 2.5.2 Feature selection

The Minimum Redundancy Maximum Relevance (MRMR) algorithm (fsrmrmr, 2025a) was applied to rank all predictors, and the top eight features were selected for further analysis.

#### 2.5.3 Model development and validation

A range of supervised regression methods was compared, including Multiple Linear Regression, Stepwise Linear Regression, Efficient Linear Regression, Support Vector Regression (SVR), Decision Trees, Ensemble methods, Gaussian Process Regression, Kernel‐based models, and Neural Networks. All neural network models used a single hidden layer with ReLU activation and were trained for 1000 epochs; only the number of hidden units and the L2 regularization strength were varied across architectures.

Model performance was evaluated using Leave‐One‐Subject‐Out (LOSO) cross‐validation, in which the model is trained on data from eight subjects and tested on the remaining subject, iterating across all participants. This approach is well suited for small sample sizes and provides an unbiased estimate of model generalizability to new individuals. Root Mean Squared Error (RMSE) and R‐squared (*R* ^2^) were used as performance metrics.

To reduce the risk of overfitting—particularly for nonlinear models such as neural networks—we conducted additional analyses using L2 regularization (*λ* = 0.05 and 0.1) and by varying network capacity through different hidden‐layer sizes (10, 50, and 100 units). Regularization strength and network size were selected based on LOSO performance. The final model was chosen as the configuration providing the best balance between predictive accuracy and generalizability.

#### 2.5.4 Model optimization

The best‐performing model was further refined using the Relief Feature Selection Algorithm (RReliefF) (MathWorks, Inc, 2025b), to reduce predictor and improve model stability and interpretability.

## 3 RESULTS

### 3.1 Demographic characteristics

The demographic characteristics of the participants are summarized in Table 2. All participants had normal body weight (BMI 18.5–24.9 kg/m^2^) (WHO, n.d.) and ventilatory function, with no abnormalities detected by spirometry.

**TABLE 2:** Demographic characteristics of participants (*n* = 9).

Variable	Mean ± SD
Age (years)	24.38 ± 3.33
Women (*n*)	4 (44.4%)
Weight (kg)	63.70 ± 11.76
Height (cm)	169.44 ± 8.39
BMI (kg/m^2^)	22.06 ± 2.71
Muscle mass (kg)	27.78 ± 6.29
Fat mass (kg)	14.00 ± 5.54
FVC (L)	3.87 ± 0.66
VEF_1_ (L)	3.61 ± 0.78
VEF_1_/FVC (%)	92.81 ± 8.88

*Note*: Data are presented as Mean ± SD.

Abbreviations: BMI, body mass index; FVC, forced vital capacity; VEF_1_, forced expiratory volume in 1 s; VEF_1_/FVC, forced expiratory volume in 1 s to forced vital capacity ratio.

### 3.2 Resting state

At rest, minute ventilation (VE), oxygen consumption (*VO*_2_), and metabolic equivalents (METS) increased at HA60h compared to SL, while stroke volume (SV) decreased. End‐tidal carbon dioxide pressure (*PETCO*_2_) was lower at HA60h compared to HA12h, accompanied by a higher ventilatory equivalent for carbon dioxide (*VE*/*VCO*_2_) and end‐tidal oxygen pressure (*PETO*_2_). Arterial oxygen saturation (*S*p*O*_2_) was consistently reduced at both HA time points compared to SL (Table 3). Individual subject data are presented in Appendix Tables A1 and A2.

**TABLE 3:** Resting physiological measurements (mean values from the final 30 s before warm‐up) at sea level (SL), after 12 h at high altitude (HA12h), and after 60 h at high altitude (HA60h).

Variable	SL	HA12h	HA60h	*p*‐value
RR (bpm)	12.6 ± 4.3	15.1 ± 5.1	18.6 ± 8.9	>0.05
VE (L)	13.4 ± 5.1	13.6 ± 6.4	16.9 ± 6.9	0.027*
*VO*_2_ (*ml/kg/min*)	5.45 ± 1.57	6.27 ± 2.40	6.79 ± 2.58	0.027*
*VCO*_2_ (*L/min*)	0.32 ± 0.11	0.34 ± 0.14	0.36 ± 0.12	>0.05
RER	0.85 ± 0.09	0.88 ± 0.04	0.88 ± 0.02	>0.05
HR (bpm)	81.5 ± 15.6	92.6 ± 14.8	93.7 ± 14.7	>0.05
*VE/VCO*_2_	37.4 ± 5.3	36.3 ± 6.2	40.1 ± 8.5	0.039***
*VO*_2_/*HR* (*ml/beat*)	4.2 ± 1.3	4.3 ± 1.9	4.6 ± 1.9	>0.05
METS	1.57 ± 0.44	1.79 ± 0.69	1.95 ± 0.74	0.027*
*PETCO*_2_ (*mmHg*)	28.5 ± 4.8	30.4 ± 4.7	27.0 ± 5.2	0.003***
*PETO*_2_ (*mmHg*)	109.2 ± 7.3	69.0 ± 5.07	72.5 ± 4.9	0.003*,**,***
SBP (mmHg)	125.7 ± 17.4	130.6 ± 26.7	132.8 ± 23.3	>0.05
DBP (mmHg)	80.1 ± 14.3	86.5 ± 12.1	85.9 ± 16.1	>0.05
SV (ml)	73.4 ± 26.3	64.1 ± 23.2	52.2 ± 13.9	0.027*
CO (L/min)	6.8 ± 2.6	8.2 ± 3.1	5.7 ± 1.3	>0.05
TPR (mmHg.s/m)	0.8 ± 0.3	0.8 ± 0.2	1.0 ± 0.2	>0.05
SpO_2_ (%)	95.9 ± 1.8	90.0 ± 2.8	91.1 ± 3.2	0.016** 0.031*

*Note*: Data are presented as Mean ± SD.

Abbreviations: CO, cardiac output; DBP, diastolic blood pressure; HR, heart rate; METS, metabolic equivalents; *PETCO*_2_, end‐tidal carbon dioxide pressure; *PETO*_2_, end‐tidal oxygen pressure; RER, respiratory exchange ratio; RR, respiratory rate; SBP, systolic blood pressure; *S*p*O*_2_, arterial oxygen saturation; SV, stroke volume; TPR, total peripheral resistance; *VCO*_2_, carbon dioxide production; VE, minute ventilation; *VE*/*VCO*_2_, ventilatory equivalent for carbon dioxide; *VO*_2_, oxygen consumption; *VO*_2_/*HR*, pulse of oxygen.

* *p <* 0.05 between SL vs. HA60h.

** *p <* 0.05 between SL vs. HA12h.

*** *p <* 0.05 between HA12h vs. HA60h.

The hypoxic ventilatory response (HVR) at HA12h (1.244 ± 1.911 L/min/kg, *p* = 0.023) and HA60h (0.954 ± 0.847 L/min/kg, *p* = 0.007) was statistically different from zero.

### 3.3 Exercise performance

**FIGURE 2:**
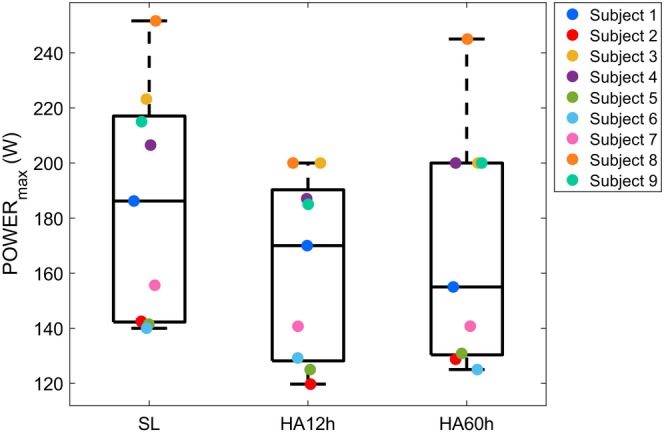
Maximal power output (POWER_max_) for each subject (*n* = 9) at sea level (SL), after 12 h at high altitude (HA12h), and after 60 h at high altitude (HA60h). Individual colored points represent POWER_max_ values for each subject, calculated as the mean power during the last 30 s of the maximal exercise test. Boxplots show the median and interquartile range (IQR), with whiskers extending to the most extreme data points within 1.5 × IQR or to the minimum/maximal values when no further points fall within that range.

**FIGURE 3:**
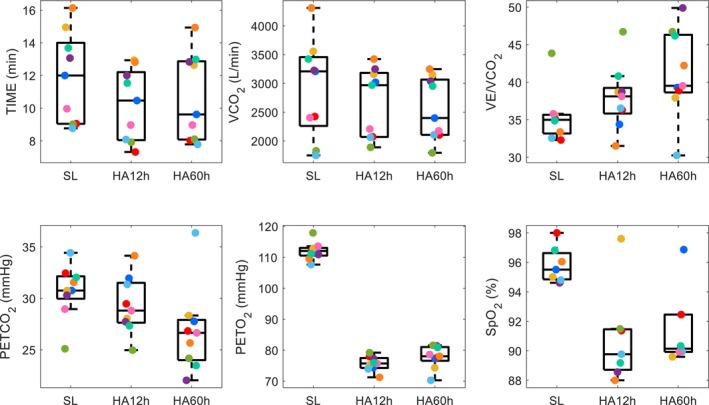
Statistically significant variables at maximal exercise (mean values from the final 30 s before fatigue) at sea level (SL), after 12 h at high altitude (HA12h), and after 60 h at high altitude (HA60h). Variables shown include total exercise time (TIME), carbon dioxide production (*VCO*_2_), ventilatory equivalent for carbon dioxide (*VE*/*VCO*_2_), end‐tidal carbon dioxide pressure (*PETCO*_2_), end‐tidal oxygen pressure (*PETO*_2_), and arterial oxygen saturation (*SpO*_2_). Individual colored points represent data from each subject. Boxplots display the median and interquartile range (IQR), with whiskers extending to the most extreme data points within 1.5 × IQR or to the minimum/maximum values when no further points fall within that range.

At maximal effort (final 30 s before fatigue), *VE*/*VCO*_2_ increased at both HA12h and HA60h compared to SL, while maximal power output (POWER), total exercise time, *PETO*_2_, and *S*p*O*_2_ were significantly reduced (Figure 2). At HA60h, *VCO*_2_ and *PETCO*_2_ were also lower compared to SL (Figure 3 and Table 4). *VO*_2max_ remained statistically unchanged across conditions. Individual subject data are provided in the Appendix (Table A3).

**TABLE 4:** Maximal exercise measurements (final 30 s before fatigue) at sea level (SL), high altitude after 12 h of arrival (HA12h) and high altitude after 60 h of arrival (HA60h).

Variable	SL	HA12h	HA60h	*p*‐value
POWER_max_	184.6 ± 41.6	161.8 ± 33.1	169.4 ± 42.8	0.003* 0.003**
Time (*min*)	11.8 ± 2.7	10.2 ± 2.2	10.6 ± 2.6	0.003* 0.003**
RR_max_ (b*pm*)	47.7 ± 7.6	46.2 ± 7.8	47.9 ± 9.8	>0.05
VE_max_ (*L*)	105.4 ± 27.6	104.3 ± 19.6	109.7 ± 31.3	>0.05
*VO*_2max_ (*ml/kg/min*)	38.3 ± 6.8	36.8 ± 6.8	35.9 ± 6.3	>0.05
*VCO*_2max_(*L/min*)	2.9 ± 0.8	2.6 ± 0.6	2.5 ± 0.5	0.039*
RER_max_	1.18 ± 0.08	1.14 ± 0.05	1.13 ± 0.07	>0.05
HR_max_ (bpm)	184.9 ± 12.2	182.3 ± 12.5	184.2 ± 12.2	>0.05
*VE*/*VCO*_2max_	35.3 ± 3.4	37.9 ± 4.2	41.2 ± 5.8	0.019* 0.011**
*VO*_2_/*HR*_max_	13.2 ± 3.8	12.9 ± 3.1	12.2 ± 2.8	>0.05
METS_max_	10.8 ± 2.0	10.5 ± 1.9	10.1 ± 1.8	>0.05
*PETCO*_2max_ (*mmHg*)	30.7 ± 2.5	29.3 ± 2.7	26.8 ± 4.1	0.011*
*PETO*_2max_ (*mmHg*)	112.03 ± 2.8	75.65 ± 2.3	77.80 ± 3.6	0.003* 0.003**
*S*p*O*_2max_ (%)	95.8 ± 1.2	90.8 ± 3.2	91.5 ± 2.8	0.024** 0.041*

*Note*: Values are Mean ± SD.

Abbreviations: HR, heart rate; METS, metabolic equivalents; *PETCO*_2_, end‐tidal carbon dioxide pressure; *PETO*_2_, end‐tidal oxygen pressure; RER, respiratory exchange ratio; RR, respiratory rate; *S*p*O*_2_, arterial oxygen saturation; *VCO*_2_, carbon dioxide production; VE, minute ventilation; *VE*/*VCO*_2_, ventilatory equivalent for carbon dioxide; *VO*_2_, oxygen consumption; *VO*_2_/*HR*, pulse of oxygen.

* *p <* 0.05 between SL vs. HA60h.

** *p <* 0.05 between SL vs. HA12h.

Additionally, resting lactate levels showed a significant increase at HA12h compared to SL, with a further non‐significant upward trend at HA60h. During maximal efforts, lactate peaked at HA12h but without reaching statistical significance (Table 5).

**TABLE 5:** Pre and Post exercise lactate levels at sea level (SL), high altitude after 12 h of arrival (HA12h) and high altitude after 60 h of arrival (HA60h).

Status	SL	HA12h	HA60h	*p*‐value
Rest	2.2 ± 1.3	2.8 ± 1.0	3.2 ± 1.3	0.046+
Exercise	10.9 ± 3.0	11.9 ± 3.0	10.6 ± 2.2	>0.05

*Note*: The results are shown as Mean ± SD: *p <* 0.05 between SL vs. HA12h.

### 3.4 24‐h blood pressure monitoring

SBP, DBP, and MAP were higher during both wakefulness and sleep at HA compared to SL. No significant differences were observed in nocturnal blood pressure dip across conditions (Table 6).

**TABLE 6:** Blood pressure at sea level (SL), high altitude after 12 h of arrival (HA12h) and high altitude after 60 h of arrival (HA60h).

Status	SL	HA12h	HA60h	*p*‐value
SBP Vigil (mmHg)	121.2 ± 13.2	125.7 ± 16.7	129.2 ± 13.8	0.019*
SBP Sleep (mmHg)	108.1 ± 12.4	109.4 ± 13.6	113.6 ± 13.1	0.011* 0.019***
SBP 24 h (mmHg)	118.1 ± 6.4	121.0 ± 9.0	124.9 ± 8.8	0.039*
DBP Vigil (mmHg)	69.7 ± 12.1	76.4 ± 16.3	76.4 ± 10.2	0.003** 0.003*
DBP Sleep (mmHg)	57.8 ± 9.5	61.9 ± 10.8	63.9 ± 8.0	0.007*
DBP 24 h (mmHg)	67.0 ± 4.6	71.8 ± 7.2	73.1 ± 3.6	0.007** 0.003*
MAP Vigil (mmHg)	86.8 ± 11.4	92.8 ± 14.4	93.9 ± 9.8	0.039** 0.003*
MAP Sleep (mmHg)	74.4 ± 9.5	77.7 ± 11.0	80.5 ± 8.4	0.007*
MAP 24 h (mmHg)	84.1 ± 12.2	90.4 ± 11.1	88.6 ± 15.1	0.039*,***
DIP (mmHg)	14.3 ± 6.3	17.8 ± 6.3	13.3 ± 5.3	>0.05

*Note*: The results are shown as Mean ± SD.

Abbreviations: DBP, diastolic blood pressure; MAP, mean arterial pressure; SBP, systolic blood pressure.

* *p <* 0.05 between SL vs. HA60h.

** *p <* 0.05 between SL vs. HA12h.

*** *p <* 0.05 between HA12h vs. HA60h.

### 3.5 Modeling

To predict performance decline under hypoxic conditions, we used the change in maximal power output (∆POWER_max_) achieved during the incremental test as a sensitive indicator of decrements in aerobic performance. The delta (∆) was calculated as the difference between HA and SL values.

Using ∆*POWER*max at HA12h as the target variable, the MRMR feature selection algorithm identified eight key predictors from SL measurements: *VO*_2_ */HR*_max_, *VE*/*VCO*_2max_, RER_max_, RR_max_, *PETCO*_2max_, and HR, METS, and *VE*/*VCO*_2_ at rest. These predictors were used to train multiple regression models under LOSO cross‐validation (Table 7). Applying the Relief feature selection algorithm, the top two predictive features were identified as *VO*_2_ */HR*_*max*_ and resting *VE*/*VCO*_2_.

**TABLE 7:** Validation performance of all trained models. Summary of validation metrics (RMSE, MSE, *R* ^2^, and MAE) and key hyperparameters for all trained models evaluated using Leave‐One‐Subject‐Out (LOSO) cross‐validation.

Model	RMSE	MSE	*R* ^2^	MAE	Hyperparameters	
					*L1 size*	*λ*
MLR	9.98	99.57	0.40	8.09		
StepLR	9.62	92.46	0.44	7.81		
EffLin	9.94	98.87	0.41	7.50		
SVR	5.33	28.38	0.83	4.11		
Tree	12.89	166.24	0.00	9.08		
Ensemble	12.78	163.45	0.02	9.26		
GPR	10.94	119.73	0.28	5.00		
Kernel	11.66	136.01	0.18	8.26		
NN	3.91	15.33	0.91	3.23	100	0
NN	4.58	20.99	0.87	3.40	50	0
NN	4.82	23.26	0.86	3.86	10	0
NN	5.35	28.64	0.83	4.64	100	0.05
NN	5.35	30.79	0.81	4.71	50	0.05
NN	**5.65**	**31.95**	**0.81**	**5.02**	**10**	**0.05**
NN	5.34	28.55	0.83	4.51	100	0.1
NN	6.35	40.35	0.76	5.53	50	0.1
NN	6.93	48.05	0.71	5.70	10	0.1

*Note*: All models were trained using the two most predictive features identified by the Relief algorithm: *VO*_2_ */HRmax* and resting *VE*/*VCO*_2_. For neural networks, the table reports the number of hidden units (L1 Size) and L2 regularization strength (λ). The model selected for interpretation is highlighted in bold.

Abbreviations: EffLin, Efficient Linear Regression; GPR, Ensemble methods, Gaussian Process Regression; Kernel, kernel regression; MLR, Multiple linear Regression; NN, neural networks; StepLR, Stepwise Linear Regression; SVR, Support Vector Regression; Tree, decision trees.

Several modeling approaches were compared, including linear, tree‐based, kernel, and neural network regressions (Table 7). Linear models performed poorly (*R* ^2^ = 0.40–0.44; RMSE≈9.6–10 W), indicating that the relationship between SL physiology and early altitude‐induced performance loss is nonlinear and not adequately captured by linear relationships.

Nonlinear models substantially improved predictive performance. Support Vector Regression (SVR) achieved *R* ^2^ = 0.83 (RMSE = 5.33 W), while the best‐performing neural network—a wide model with 100 hidden units and no regularization—reached *R* ^2^ = 0.91 (RMSE = 3.91 W). However, this unregularized architecture involved a large number of parameters relative to the sample size (*n* = 9), raising concerns about overfitting. We therefore prioritized model parsimony and generalizability. Among the regularized architectures, a compact neural network with a single hidden layer of 10 units and L2 regularization (*λ* = 0.05) provided the best balance between predictive accuracy and model simplicity (*R* ^2^ = 0.81, RMSE = 5.65), performing comparably to SVR while enabling clearer physiological interpretation of the nonlinear response surface. This regularized neural network was selected as the final model for interpretation.

**FIGURE 4:**
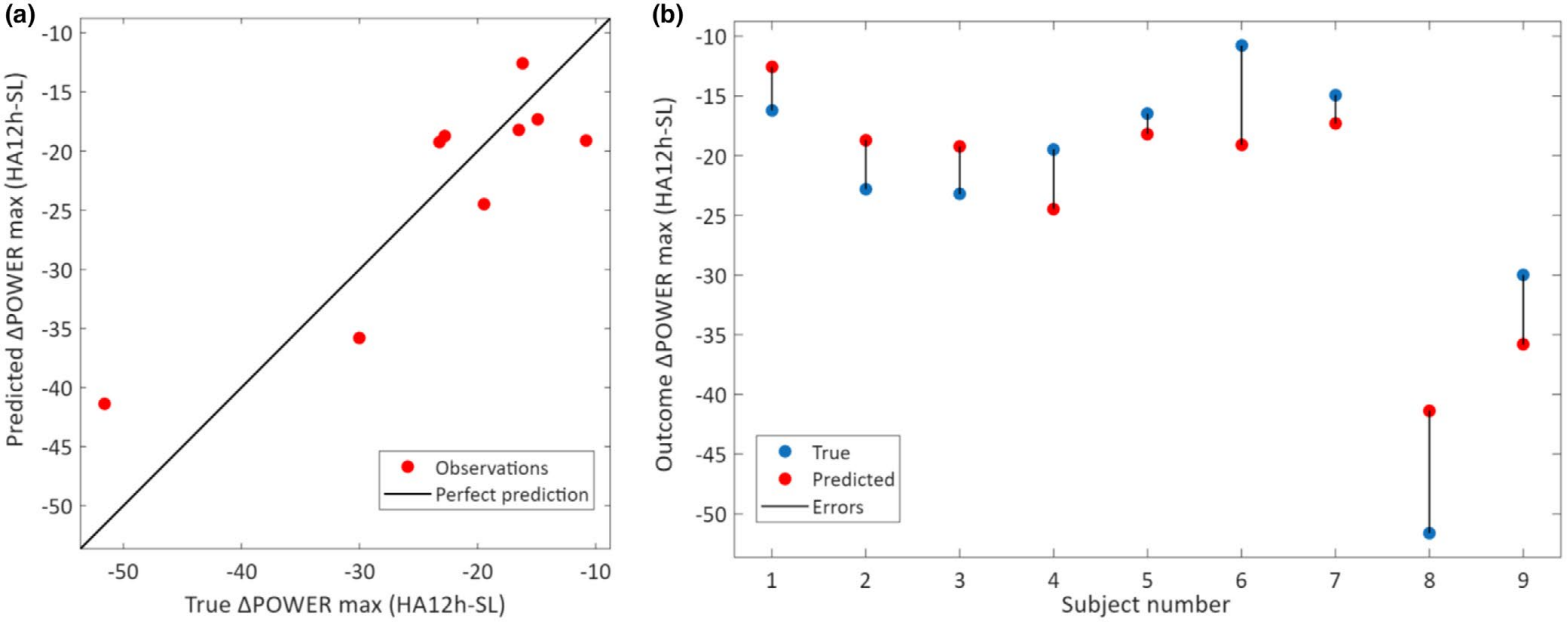
(a) Validation plots for the selected neural network model predicting ∆POWER_max_ at HA12h using SL predictors (*R* ^2^ = 0.81, RMSE = 5.65). (b) Individual LOSO predictions showing true vs. predicted values for each subject; black lines indicate prediction errors.

Agreement between predicted and observed values for the selected model is shown in Figure 4a. At the individual level, LOSO predictions were consistent across participants, with no single subject disproportionately influencing overall accuracy. Figure 4b shows the observed and predicted ∆POWER_max_ values for each subject, confirming that prediction errors were small and uniformly distributed. This supports the conclusion that the model captures a true generalizable relationship rather than isolated well‐predicted cases.

All analysis code and trained models are publicly available at: https://github.com/QuantitativePhysiologyLabUC/HypoxiaPerformancePredictor.git.

In contrast, modeling ∆POWER_max_ at HA60h yielded poor predictive performance. The best model was Efficient Linear Regression with three features selected by Relief: resting *VE*/*VCO*_2_, resting METS, and RER_max_ achieved only *R* ^2^ = 0.32 and a nearly flat prediction curve. This indicates that no meaningful relationship between SL predictors and performance loss could be established for this later time point (results shown in Appendix Table A4).

## 4 DISCUSSION

The aim of this study was twofold: (i) to examine the effects of short‐term acclimatization to HA exposure on cardiorespiratory, autonomic, and metabolic responses both at rest and during incremental exercise, and (ii) to develop predictive models capable of forecasting exercise performance decline at HA using baseline measurements collected at SL.

### 4.1 Cardiorespiratory, autonomic and metabolic adaptations to short‐term high‐altitude exposure

Our findings indicate that a 60‐h period is insufficient to achieve full acclimatization, consistent with previous reports suggesting that longer durations are required (Burtscher et al., 2022). This contrasts with studies proposing that early ventilatory acclimatization can be induced within 1–2 days (Muza et al., 2010) or that a rest period of 2–3 days at high altitude is sufficient to promote acclimatization (Sharma et al., 2023).

However, distinct physiological differences emerged between 12 and 60 h of HA exposure. At HA12h, we observed an increased hypoxic ventilatory response (HVR), which reflects a compensatory mechanism to mitigate the effects of the environmental O_2_ deficit that causes a reduction in arterial oxygen saturation (*S*p*O*_2_) and consequent tissue‐level hypoxia (Alvarez‐Araos et al., 2024; Fabries et al., 2022; Richalet et al., 2024; Tymko et al., 2023).

By HA60h, HVR remained elevated, and resting minute ventilation (VE) significantly increased compared to SL. The concurrent increase in *VE*/*VCO*_2_ and decline in *PETCO*_2_ at this point suggest that regulatory mechanisms beyond chemoreceptor‐driven HVR may be acting at this stage of acclimatization.

A recent review by Tymko et al. (Tymko et al., 2023) examined the effects of hypoxemia on muscle sympathetic nerve activity (MSNA), differentiating between acute exposure (<12 h) and chronic exposure (>12 h) to high‐altitude environments. The authors reported that MSNA burst frequency (BF) – a temporal indicator of sympathetic discharge‐ was 4–5 times higher in chronic hypoxemia than in acute hypoxemia. Likewise, MSNA burst incidence (BI) – which reflects central sympathetic drive‐ was approximately two‐fold higher with prolonged exposure. These findings underscore the critical role of exposure duration in determining the extent of sympathoexcitation.

In acute hypoxemia, sympathetic activation is primarily mediated by peripheral chemoreceptors. However, during more prolonged exposure, additional inputs from arterial baroreceptors (responding to hypovolemia and increased hemoglobin concentration) and pulmonary baroreceptors (responding to elevated pulmonary arterial resistance and pressure) likely contribute to the sustained elevation of sympathetic outflow observed at HA (Simpson et al., 2021). Interestingly, even when peripheral chemoreflex drive is experimentally eliminated, MSNA responses at HA appear only modestly eliminated, suggesting central regulatory mechanisms, such as those governed by the nucleus of the solitary tract, are involved in autonomic integrations and ventilatory drive during hypoxic exposure (Bourdillon et al., 2018; Simpson et al., 2021; Tymko et al., 2023).

Robbins (Robbins, 2007) has further proposed that ventilatory adaptations at this stage may be linked to renal compensation mechanisms and the gradual restoration of pH balance. Tymko et al. (Tymko et al., 2023) also emphasize the role of chronic respiratory alkalosis and low bicarbonate levels in shaping ventilatory efficiency. The slower homeostatic adjustments are unlikely to be present at 12 h, potentially explaining the elevated resting lactate observed at HA12h, which returned to lower levels at HA 60 h. Continued high‐resolution monitoring during the early phase of HA exposure is essential to accurately understand the full time course of acclimatization.

At rest, *PETCO*_2_ and *S*p*O*_2_ were consistently lower at HA compared to SL. During maximal‐intensity exercise – where additional compensatory mechanisms are activated to meet oxygen demands (Townsend et al., 2017)‐ we observed increases in *VE*/*VCO*_2max_ and reductions in *PETO*_2max_, along with decreased exercise duration and POWER_max_. Although *VO*_2max_ remained statistically unchanged, suggesting that participants maintained aerobic metabolism, this did not prevent performance. The maintenance of *VO*_2max_ is likely due to rapid compensatory mechanisms ensuring adequate oxygen delivery to the tissues, such as increased ventilation and redistribution of blood (Alvarez‐Araos et al., 2024). In our study, the mean of *S*p*O*_2_ remained above 90% at both HA time points, indicating that compensatory hyperventilation and ventilatory efficiency (increase in *VE*/*VCO*_2_) played a key role.

Importantly, the reduction in maximal power output and time to exhaustion likely involve mechanisms beyond oxygen availability. They include the activation of group II and IV afferent fibers during high‐intensity incremental exercise (Alvarez‐Araos et al., 2024; Amann et al., 2020; Gandevia, 1998; Hug & Dorel, 2009; Townsend et al., 2017), which in turn trigger two muscle fatigue‐related processes: the exercise pressor reflex influenced by muscle mechanical stimulation and metabolic signal (e.g. pH, lactate levels) (Alvarez‐Araos et al., 2024) and central inhibition of alpha motor neurons at the spinal level, which limit motor unit recruitment (Amann et al., 2020; Gandevia, 1998; Hug & Dorel, 2009).

Blood pressure (BP) response is influenced by the predominance of either vasodilatory or vasoconstrictive effects, which result from the divergent reactions of central and peripheral BP regulatory mechanisms (Richalet et al., 2024; Tymko et al., 2023). During resting conditions—both in wakefulness and over a 24‐h period—we observed a predominant acute sympathetic vasoconstrictive response, with higher SBP, along with a higher MAP in wakefulness. This finding aligns with previous observations that while acute (<12 h) hypoxia may not significantly alter MAP (Tymko et al., 2023), prolonged exposure results in MAP elevation (Tymko et al., 2023). Although BP is often considered stable in acute HA (Richalet et al., 2024), many prior studies relied on single point measurements. For instance, in a normobaric hypoxia study mimicking 4500 m of altitude, 80 healthy subjects showed no change in MAP after 12 h (Niebauer et al., 2020). But this assessment lacked 24‐h monitoring. In contrast, our study employed ambulatory blood pressure monitoring (ABPM), which is recognized as the gold standard for evaluating BP and hypertension (Hermida et al., 2015). ABPM‐based studies in normotensive subjects remain scarce. However, one study by Torlasco et al. (Torlasco et al., 2020) reported significant increases in SBP and DBP during wakefulness, sleep, and across the 24‐h period after arrival at moderate altitude (2035 m), corroborating our findings at 3015 m. These results reinforce the utility of ABPM in understanding cardiovascular strain during acute and intermediate HA exposure.

### 4.2 Predictive modeling approaches for performance at acute high‐altitude

In the context of personalized medicine and efforts to identify individual susceptibility to high‐altitude‐induced performance impairment, we explored whether machine learning (ML) techniques could predict performance decline – specifically in maximal power output (POWER_max_)‐ using sea level measurements in healthy individuals. The goal was to develop a quantitative, non‐invasive method for identifying individuals likely to experience greater performance deterioration when exposed to HA.

We found that predicting ∆POWER_max_ at HA12h is feasible using SL‐derived variables, particularly those obtained during both resting and maximal phases of the CPET. The selected neural network model demonstrated strong predictive performance, with a root mean squared error (RMSE) of 5.65 W, indicating a relatively small average deviation between predicted and observed values. Importantly, simpler linear models performed significantly worse (*R* ^2^≈0.40–0.44), and although nonlinear SVR also performed well (*R* ^2^ = 0.83), the regularized neural network offered the best balance between accuracy and model parsimony. The consistency of per‐subject LOSO predictions further supports the model's generalizability and reduces concerns about overfitting.

Previously, mathematical models have been used to estimate power‐related variables such as critical power (CP) and work above CP (W′), including polynomial functions‐based models like the work‐prime balance (W'BAL), which simulate fatigue dynamics during high‐intensity interval training (HIIT) under moderate hypoxia (e.g. 2250 m) with high accuracy (*R* ^2^ = 0.99). However, to our knowledge, no prior work has attempted to predict exercise performance decline under hypoxic conditions based on cardiorespiratory variables measured at sea level (Townsend et al., 2017). This represents a meaningful conceptual shift: rather than modeling exercise dynamics during hypoxia, we attempt to anticipate who will be more affected by acute hypoxic stress before exposure occurs.

Recent ML applications in altitude physiology have focused on predicting hypoxia‐related cognitive impairments (Liu et al., 2022), acute mountain sickness (Wei et al., 2022), and myocardial ischemia (Chen et al., 2024). Additionally, real‐time physiological monitoring wearable sensors and ML algorithms have been implemented in aviation to detect early signs of hypoxia, enhancing flight safety (Snider et al., 2022). Over the past two decades, neural networks and deep learning models have seen increasing application in healthcare and sports performance (Trujillano et al., 2004). In comparison to traditional models such as logistic regression or linear regression, deep learning models leverage neural networks for their ability to learn complex patterns from data, offering greater flexibility and showing promising results even with relatively small datasets (Choi et al., 2016; Rasmy et al., 2018; Trujillano et al., 2004).

Our results suggest that integrating ML into HA performance prediction has potential utility in clinical, occupational, and sports domains. Future research should expand sample sizes and refine algorithms to improve generalizability, enabling personalized acclimatization strategies for diverse populations.

A limitation of this study was the absence of dietary control. However, this decision allowed us to simulate real‐world conditions more accurately reflecting the typical behavior of individuals exposed to HA environments. Despite the small sample size (*n* = 9), the study's robustness stems from its repeated‐measures design, multimodal monitoring, and inclusion of both male and female participants. Each subject underwent four assessments, including 24‐h physiological recordings at SL and HA. The first SL test was used for familiarization and excluded from analysis, while the subsequent SL, HA12h, and HA60h tests formed the analytical dataset.

We acknowledge that the predictive model was developed as a pilot with limited generalizability. Nevertheless, it establishes a foundation for future work aimed at reducing the frequency of in‐field monitoring and optimizing predictor selection through scalable, data‐driven approaches.

A short 60‐h exposure to high altitude is insufficient for full physiological acclimatization. However, meaningful changes in respiratory, metabolic, and autonomic responses were observed between 12 and 60 h of exposure. Notably, our study demonstrates that deterioration in exercise performance at HA can be anticipated using parameters measured at SL.

These findings offer novel insights into the early stages of acclimatization and highlight key physiological determinants of HA‐induced performance decline. Furthermore, they support the use of machine learning as a promising tool for predicting individual responses to hypoxic stress prior to exposure.

Understanding these responses is essential for optimizing physical performance, reducing health risks, and informing targeted acclimatization and pre‐acclimatization strategies. These results have practical implications for athletes, clinicians, and professionals operating in high‐altitude environments.

## AUTHOR CONTRIBUTIONS

V.P. contributed to the conception and design of the study, data acquisition, data analysis and interpretation, and drafting and critical revision of the manuscript. M.R.‐F. contributed to the conception and design of the study, data analysis and interpretation, and to drafting and critically revising the manuscript. D.C.A. contributed to drafting and critically revising the manuscript. S.L. and D.C. contributed to data acquisition. All authors approved the final version of the manuscript and agree to be accountable for all aspects of the work. Any questions regarding the accuracy or integrity of the study should be directed to the corresponding author.

## FUNDING INFORMATION

This work was supported by ANID under grants ACT210083, Fondecyt 1230844, ANID‐Subdirección de Capital Humano/Doctorado Nacional/2023–21230255, and CIA250016 (CPS‐RTC). DCA was supported by U.S. grant AFOSR‐SOARD FA9550‐24‐1‐0248 and ANID NAM NAM24I0095.

## CONFLICT OF INTEREST STATEMENT

The authors declare that they have no competing interests or conflicts of interest related to this work.

## ETHICS STATEMENT

All procedures involving human participants were conducted in accordance with the ethical standards of the institutional research committee and with the principles of the Declaration of Helsinki. Written informed consent was obtained from all participants prior to their inclusion in the study. The study protocol was reviewed and approved by the Ethics Committee of the Pontificia Universidad Católica de Chile (approval number 220906004). All data were collected and analyzed anonymously. No individually identifiable information or images of participants are included in this manuscript.

## Data Availability

The data and predictive model generated during the current study are available in Hypoxia Performance Predictor Repository, at the following link: https://github.com/QuantitativePhysiologyLabUC/HypoxiaPerformancePredictor.git.

## APPENDIX A

**TABLE A1:** Individual subject data in resting state by ergoespirometry.

Variable	Condition	Subject								
		1	2	3	4	5	6	7	8	9
VO2/HR	SL	4.99	2.90	5.62	5.43	3.98	1.67	3.50	4.80	5.80
	HA12h	5.25	3.22	9.18	4.04	3.45	3.16	2.59	3.66	4.94
	HA60h	7.34	2.26	7.82	3.65	4.09	3.16	5.23	3.03	4.86
HR	SL	70.50	73.75	84.00	77.00	79.57	121.00	83.86	73.00	71.60
	HA12h	69.25	101.00	78.50	120.80	93.50	102.46	89.71	93.29	85.11
	HA60h	77.43	91.43	72.50	121.44	104.08	90.91	95.15	87.75	102.73
METS	SL	1.96	1.03	2.25	1.73	1.90	0.97	1.51	1.18	1.60
	HA12h	1.78	1.55	3.45	1.96	1.94	1.52	1.17	1.11	1.62
	HA60h	2.80	0.98	2.72	1.81	2.52	1.34	2.49	0.90	1.95
VE/VCO2	SL	41.06	34.83	44.30	32.70	39.13	43.87	39.25	30.00	31.52
	HA12h	49.63	33.69	42.17	29.42	34.90	35.66	37.94	32.71	31.07
	HA60h	55.39	42.77	51.13	32.48	40.58	39.70	35.66	32.80	30.70
RER	SL	‐	0.86	‐	0.73	0.76	‐	0.98	0.89	0.88
	HA12h	0.87	0.92	0.96	0.90	0.84	0.72	0.89	0.90	0.81
	HA60h	0.81	0.88	0.94	0.90	0.84	0.83	0.91	0.90	0.88
VCO2	SL	454.46	185.18	547.33	299.50	243.76	221.05	286.57	311.77	364.35
	HA12h	324.53	298.88	689.01	437.08	269.59	233.41	205.26	305.69	340.93
	HA60h	459.69	182.48	534.99	389.40	354.61	225.30	447.45	241.24	448.32
VO2	SL	6.85	3.51	7.87	5.96	6.67	3.34	5.35	4.04	5.59
	HA12h	6.25	5.44	12.05	6.94	6.73	5.38	4.09	3.87	5.67
	HA60h	9.78	3.45	9.44	6.31	8.79	4.71	8.75	3.07	6.85
VE	SL	18.07	9.30	24.95	12.92	10.37	10.03	12.19	9.93	12.86
	HA12h	16.02	11.47	29.81	13.45	11.04	10.20	8.66	10.58	11.71
	HA60h	26.90	8.92	28.37	14.34	16.29	17.03	17.73	8.21	15.18
RR	SL	13.23	16.02	10.11	14.76	13.94	5.07	12.18	8.39	19.90
	HA12h	12.70	17.91	9.03	9.56	15.63	26.13	12.61	15.45	17.13
	HA60h	14.18	14.69	9.73	15.28	24.83	36.76	24.43	7.86	20.28
PETCO2	SL	26.00	31.63	23.17	29.33	26.57	24.00	24.86	35.25	36.50
	HA12h	22.75	32.44	25.25	35.40	30.88	27.77	28.29	35.86	35.33
	HA60h	19.86	26.86	19.33	32.88	25.75	26.10	27.33	33.75	32.00
PETO2	SL	117.29	103.63	118.33	103.00	107.67	116.67	114.43	100.75	101.40
	HA12h	77.50	68.78	75.50	65.40	67.86	67.50	71.86	64.29	62.33
	HA60h	78.43	73.86	81.17	68.56	73.08	72.10	72.50	65.75	67.82
Lactate	SL	3.75	0.75	‐	4.30	1.55	1.30	1.40	2.50	‐
	HA12h	4.0	2.40	1.10	4.05	2.25	1.75	2.45	3.90	3.65
	HA60h	3.95	5.70	2.60	3.40	1.55	1.95	3.25	4.45	2.25
SPO2	SL	95.0	97.6	98.4	93.8	93.7	96.0	‐	97.8	95.3
	HA12h	84.0	‐	93.0	90.8	91.51	89.9	‐	90.8	89.9
	HA60h	94.2	94.1	95.0	‐	89.0	86.8	90.0	‐	88.7

Abbreviations: HA12h, high altitude after 12 h of arrival; HA60, high altitude after 60 h of arrival; HR, heart rate; Lactate, blood lactate concentration; METS, metabolic equivalents; PETCO2, end‐tidal carbon dioxide pressure; PETO2, end‐tidal oxygen pressure; RER, respiratory exchange ratio; RR, respiratory rate; SL, sea level; SPO2, peripheral oxygen saturation; VCO2, carbon dioxide production; VE/VCO2, ventilatory equivalent for carbon dioxide; VE, minute ventilation; VO2/HR, oxygen pulse; VO2, oxygen uptake.

**TABLE A2:** Individual subject data in resting state by beat‐to‐beat‐hemodynamic.

Variable	Condition	Subject								
		1	2	3	4	5	6	7	8	9
SBP	SL	122.38	116.06	141.14	133.16	105.32	152.96	136.71	98.15	126.12
	HA12h	81.34	141.95	171.48	133.61	114.96	159.94	134.01	111.61	126.78
	HA60h	100.56	164.89	160.88	153.06	135.90	136.64	121.55	110.48	111.32
DBP	SL	73.15	59.96	83.39	73.73	69.93	96.37	101.13	69.10	94.24
	HA12h	67.82	84.87	104.29	86.00	78.55	104.04	79.66	80.10	93.78
	HA60h	68.30	108.26	95.43	100.96	68.31	93.19	80.08	63.88	94.75
SV	SL	84.16	91.72	117.73	95.55	43.05	52.69	37.70	69.10	68.99
	HA12h	34.31	79.89	109.74	71.46	44.38	55.64	52.56	80.10	49.40
	HA60h	60.91	50.08	79.32	48.38	49.90	44.68	41.32	63.88	31.91
CO	SL	5.40	9.03	11.47	6.80	3.80	9.34	3.92	6.65	4.90
	HA12h	3.95	13.44	10.35	9.99	6.10	10.68	4.54	8.38	7.25
	HA60h	6.31	7.84	5.43	5.44	6.47	4.67	4.20	7.03	4.02
TPR	SL	0.81	0.55	0.48	0.69	0.93	0.86	1.74	0.61	0.88
	HA12h	1.20	0.47	0.72	0.62	0.89	0.74	1.14	0.67	0.93
	HA60h	0.84	1.03	1.05	1.25	0.71	1.01	1.32	0.76	1.42

Abbreviations: CO, cardiac output; DBP, diastolic blood pressure; HA12h, high altitude after 12 h of arrival; HA60, high altitude after 60 h of arrival; SBP, systolic blood pressure; SL, sea level; SV, stroke volume; TPR, total peripheral resistance.

**TABLE A3:** Individual subject data in maximal exercise by ergoespirometry.

Variable	Condition	Subject								
		1	2	3	4	5	6	7	8	9
POWER	SL	186.2	142.5	223.2	206.5	141.5	140.0	155.6	251.6	215.0
	HA12h	170.0	119.7	200.0	187.0	125.0	129.2	140.7	200.0	185.0
	HA60h	155.0	128.8	200.0	200.0	130.9	125.0	140.8	245.0	200.0
Time	SL	12.00	9.05	14.95	13.06	9.01	8.76	9.96	16.13	13.68
	HA12h	10.46	7.31	12.93	12	7.91	8.08	8.96	12.81	11.53
	HA60h	9.61	8.01	12.63	12.8	8.10	7.78	8.96	14.93	12.98
VO2/HR	SL	14.0	10.8	15.2	14.3	8.4	8.2	11.8	19.8	17.1
	HA12h	14.8	9.7	15.8	14.5	8.6	9.2	11.6	16.1	15.8
	HA60h	12.1	11.3	16.5	13.8	7.8	9.1	11.0	15.3	13.8
HR	SL	188.3	167.4	183.6	196.8	188.9	199.7	166.7	195.8	177.5
	HA12h	178.3	167.3	181.2	200.1	186.0	199.1	163.8	187.8	177.5
	HA60h	175.0	173.5	184.1	204.7	184.5	196.5	166.1	193.1	180.5
METS	SL	13.2	8.4	13.3	11.4	9.4	7.8	10.1	12.7	11.7
	HA12h	13.0	7.7	13.6	11.9	9.6	8.8	9.6	9.9	10.8
	HA60h	10.4	9.3	14.4	11.5	8.4	8.5	9.2	9.7	9.6
VE/VCO2	SL	35.0	32.3	35.6	35.1	43.9	32.5	35.8	33.4	34.9
	HA12h	34.4	36.3	38.7	38.7	46.7	36.5	38.1	31.5	40.8
	HA60h	39.3	38.9	37.9	49.9	46.7	30.2	39.5	42.2	46.2
RER	SL	1.2	1.3	1.3	1.1	1.1	1.1	1.2	1.1	1.1
	HA12h	1.1	1.3	1.1	1.1	1.2	1.2	1.2	1.1	1.1
	HA60h	1.1	1.1	1.0	1.1	1.3	1.2	1.2	1.1	1.2
VCO2	SL	320.7	242.2	355.9	322.9	182.4	174.1	240.6	431.4	342.5
	HA12h	301.8	207.8	316.0	324.5	188.5	206.1	220.9	342.8	296.6
	HA60h	239.6	210.5	314.8	303.1	179.8	210.5	217.3	324.9	295.2
VO2	SL	46.11	29.41	46.63	39.89	32.99	29.07	35.17	44.51	40.93
	HA12h	45.61	27.01	47.53	41.54	33.46	30.63	33.55	34.72	37.87
	HA60h	36.55	32.68	49.79	40.40	29.54	29.72	32.25	33.93	33.61
VE	SL	115.9	80.9	131.3	114.4	85.7	60.9	89.9	147.5	122.6
	HA12h	105.6	78.6	125.9	130.3	92.4	83.5	88.2	110.8	124.1
	HA60h	98.2	84.3	126.3	156.0	86.8	67.0	88.7	140.7	140.0
RR	SL	49.7	35.9	59.2	38.0	57.1	49.7	46.2	48.7	45.6
	HA12h	44.5	33.1	56.5	44.1	58.3	50.9	41.3	42.3	45.4
	HA60h	40.0	31.8	56.4	55.6	60.8	43.0	38.5	53.2	52.2
PETCO2	SL	30.8	32.4	30.8	30.3	25.1	34.4	29.0	31.6	32.0
	HA12h	32.0	29.5	28.0	27.7	25.0	31.4	28.8	34.1	27.3
	HA60h	27.8	26.8	28.3	22.0	24.2	36.4	26.7	25.7	23.5
PETO2	SL	112.6	112.1	112.9	110.9	117.9	107.7	113.6	109.5	111.2
	HA12h	74.3	77.9	75.3	77.3	79.2	74.0	75.7	71.2	75.9
	HA60h	77.3	77.8	74.3	81.4	81.6	70.3	78.6	78.0	80.9
Lactate	SL	14.35	6.30	‐	13.60	11.00	9.60	13.30	8.35	‐
	HA12h	17.60	8	12.60	12.20	7.95	12.20	11.70	14.75	10.30
	HA60h	9.45	8.3	11.80	12.05	9.30	7.45	12.55	14.35	10.50
SPO2	SL	95.5	98.0	95.0	94.6	‐	94.7	‐	96.0	96.8
	HA12h	‐	91.3	97.6	88.5	91.4	89.7	‐	88.0	89.1
	HA60h	96.8	92.4	89.5	‐	‐	89.9	89.9	‐	90.3

Abbreviations: HA12h, high altitude after 12 h of arrival; HA60, high altitude after 60 h of arrival; HR, heart rate; Lactate, blood lactate concentration; METS, metabolic equivalents; PETCO2, end‐tidal carbon dioxide pressure; PETO2, end‐tidal oxygen pressure; POWER, potency; RER, respiratory exchange ratio; RR, respiratory rate; SL, sea level; SPO2, peripheral oxygen saturation; Time, time of exercise; VCO2, carbon dioxide production; VE, minute ventilation; VE/VCO2, ventilatory equivalent for carbon dioxide; VO2, oxygen uptake; VO2/HR, oxygen pulse.

**TABLE A4:** Validation performance of all trained models for predicting ∆POWER_max_ at HA60h. The table summarizes validation metrics (RMSE, MSE, *R* ^2^, MAE) for all evaluated models.

Model	RMSE	MSE	*R* ^2^	MAE
MLR	7.31	53.48	0.23	6.65
StepLR	8.74	76.44	−0.10	7.81
**EffLin**	**6.88**	**47.27**	**032**	**5.55**
SVM	7.63	58.28	0.16	6.02
Tree	8.33	69.33	0.00	6.01
Ensemble	8.31	69.10	0.00	587
GPR	8.91	79.37	−0.14	7.04
Kernel	7.60	57.73	0.17	5.75
NN	18.45	340.36	−3.91	15.61

*Note*: All models were trained using the three top predictive features identified by the Relief algorithm: *VE*/*VCO*_2_ and METS at rest and RER_max_. Methods include Multiple linear Regression (MLR), Stepwise Linear Regression (StepLR), Efficient Linear Regression (EffLin), Support Vector Machine (SVM), Decision Tree (Tree), Ensemble, Gaussian Process Regression (GPR), Kernel Regression (Kernel), and Neural Networks (NN). The model selected for interpretation is highlighted in bold.

Abbreviations: MAE, mean absolute error; MSE, mean squared error; *R* ^2^, *R*‐squared; RMSE, root mean squared error.
